# Evaluating the effectiveness of the National Health Insurance Fund in providing financial protection to households with hypertension and diabetes patients in Kenya

**DOI:** 10.1186/s12939-023-01923-5

**Published:** 2023-06-01

**Authors:** Robinson Oyando, Vincent Were, Hillary Koros, Richard Mugo, Jemima Kamano, Anthony Etyang, Adrianna Murphy, Kara Hanson, Pablo Perel, Edwine Barasa

**Affiliations:** 1 Health Economics Research Unit, KEMRI Wellcome Trust Research Programme, P.O.BOX 43640-00100, Nairobi, Kenya; 2 AMPATH, Eldoret, Kenya; 3 Department of Medicine, School of Medicine, College of Health Sciences, Moi University, Eldoret, Kenya; 4 Department of Epidemiology and Demography, KEMRI-Wellcome Trust Research Program, Kilifi, Kenya; 5 Department of Health Services Research and Policy, London School of Hygiene & Tropical Medicine, London, UK; 6 Department of Global Health and Development, London School of Hygiene & Tropical Medicine, London, UK; 7 Department of Non-Communicable Disease Epidemiology, London School of Hygiene and Tropical Medicine, London, UK; 8 Center for Tropical Medicine and Global Health, Oxford University, Oxford, 01540 UK

**Keywords:** Non-communicable diseases, Health expenditure, Financial risk protection, Health insurance, Kenya

## Abstract

**Background:**

Non-communicable diseases (NCDs) can impose a substantial financial burden to households in the absence of an effective financial risk protection mechanism. The national health insurance fund (NHIF) has included NCD services in its national scheme. We evaluated the effectiveness of NHIF in providing financial risk protection to households with persons living with hypertension and/or diabetes in Kenya.

**Methods:**

We carried out a prospective cohort study, following 888 households with at least one individual living with hypertension and/or diabetes for 12 months. The exposure arm comprised households that are enrolled in the NHIF national scheme, while the control arm comprised households that were not enrolled in the NHIF. Study participants were drawn from two counties in Kenya. We used the incidence of catastrophic health expenditure (CHE) as the outcome of interest. We used coarsened exact matching and a conditional logistic regression model to analyse the odds of CHE among households enrolled in the NHIF compared with unenrolled households. Socioeconomic inequality in CHE was examined using concentration curves and indices.

**Results:**

We found strong evidence that NHIF-enrolled households spent a lower share (12.4%) of their household budget on healthcare compared with unenrolled households (23.2%) (*p =* 0.004). While households that were enrolled in NHIF were less likely to incur CHE, we did not find strong evidence that they are better protected from CHE compared with households without NHIF (OR = 0.67; *p* = 0.47). The concentration index (CI) for CHE showed a pro-poor distribution (CI: -0.190, *p* < 0.001). Almost half (46.9%) of households reported active NHIF enrolment at baseline but this reduced to 10.9% after one year, indicating an NHIF attrition rate of 76.7%. The depth of NHIF cover (i.e., the share of out-of-pocket healthcare costs paid by NHIF) among households with active NHIF was 29.6%.

**Conclusion:**

We did not find strong evidence that the NHIF national scheme is effective in providing financial risk protection to households with individuals living with hypertension and/diabetes in Kenya. This could partly be explained by the low depth of cover of the NHIF national scheme, and the high attrition rate. To enhance NHIF effectiveness, there is a need to revise the NHIF benefit package to include essential hypertension and/diabetes services, review existing provider payment mechanisms to explicitly reimburse these services, and extend the existing insurance subsidy programme to include individuals in the informal labour market.

## Background

The disproportionate increase in the burden of non-communicable diseases (NCDs) in low-and middle-income countries (LMICs) poses a challenge to health systems’ performance in the area of financial protection. Financial protection is often measured by whether or not a household has experienced “catastrophic health expenditure” (CHE), defined as health expenditure that exceeds a selected income threshold [1–3]. Whilst protection against CHE through health insurance is not a guarantee [4, 5], it has been shown that uninsured NCD patients are 2–7 times more likely to incur CHE than insured patients [3]. In addition, evidence suggests the existence of socioeconomic inequalities in the utilisation of cost-effective interventions for NCDs, with the poor being disadvantaged [6–10]. A health system response to NCDs should therefore ideally include mechanisms that ensure equitable access to needed interventions that are of good quality and protect against financial risk by ensuring effective prepayment financing arrangements [11, 12].

In Kenya, NCDs account for 50% of hospitalisations and 39% of deaths [13, 14]. In addition, NCDs require long-term care and have been shown to present a major economic burden to households in Kenya [15–18]. For instance, compared to non-NCD households, households with one member with an NCD (NCD households) are twice as likely to incur CHE [15]. Furthermore, other studies conducted in Kenya have shown that diabetes and hypertension treatment costs disproportionately affect poorer households, with medicine and transport-related costs contributing to the largest share of total annual costs [16, 17]. Similarly, screening, diagnosis and treatment costs for NCDs like cancer are not affordable to a majority of Kenyan households given low income levels and inadequate prepayment arrangements [18].

To move towards universal health coverage (UHC) where every Kenyan is able to access the quality health services that they need without facing financial hardship, the government of Kenya has made a policy decision to attain this goal by expanding financial risk protection through the National Health Insurance Fund (NHIF) [19]. The NHIF is Kenya’s public health insurer with the mandate to provide health insurance cover to all Kenyans [20, 21]. Individuals enrolled in the NHIF make monthly premium contributions in the form of income-graduated statutory deductions for formal sector employees and voluntary payments of a flat rate of Kenya shillings (KES) 500 (USD 4) for individuals in the informal labour market [20]. NHIF members receive a membership card and are required to present the card to health facilities to access the NHIF benefit package. Health facilities provide health services to NHIF members and are in turn paid by the NHIF using a capitation mechanism for outpatient services, per-admission day payment for in-patient services, and case-based payments for selected services such as surgeries, and deliveries. These payments are expected to be cover the full cost of care in public facilities, who are prohibited by the contracts they sign with the NHIF from balance billing NHIF members. Private facilities are allowed to balance bill when the cost of care is deemed higher than the NHIF payment rates [22]. Population coverage with the NHIF is low, estimated to be 24% of the Kenyan population [23]. In 2015, the NHIF expanded its benefits package for members under the national scheme to include outpatient, inpatient, and specialised services that include NCDs such as diabetes and hypertension [19, 20]. This expanded benefit package was dubbed “Supa cover”. Despite the shifting epidemiological disease burden in Kenya, no study has assessed whether NHIF protects NCD households from incurring CHE. Therefore, we set out to measure the share of household expenditure that is spent on healthcare, the incidence of CHE, and the effectiveness of the NHIF national scheme in providing financial risk protection to households with hypertension and/or diabetes patients—two major NCDs in Kenya [24, 25].

## Methods

### Study setting

Kenya is a lower middle-income country with a gross domestic product (GDP) per capita of US Dollars (USD) 2,007 [26]. Kenya has a population of more than 47 million, of whom 69% reside in rural areas [27]. About 36.1% of the population lives below the international poverty line, spending less than USD 1.9 per day [28]. There are two levels of governance in Kenya: the national government and 47 semi-autonomous county governments [29]. Kenya’s health system is pluralistic with an almost equal share in the provision of healthcare services by the public and private providers [30]. The national government (Ministry of Health) is responsible for policy and regulatory roles in the health system and for overseeing service delivery in national referral hospitals while the county governments are responsible for service delivery at the county level [31]. Public sector health service delivery is organized into four levels: (a) community services - level 1 community units providing community-based demand creation activities, (b) primary health services- (level 2 dispensaries and level 3 - health centers), (c) county referral services (level 4 and 5 hospitals) and (d) national referral services (level 6 hospitals) [31]. The health system is financed through out-of-pocket (OOP) payments by households, government (national and county) tax revenues, donors, member contributions from NHIF and private insurance companies [32, 33]. In the calendar year 2020, current health expenditure as a percentage of gross domestic product was 4.3%, current health expenditure per capita in US$ was 83.4 while the proportion of current health expenditure financed through OOP payments was 24.1% [34]. Regarding NCD financing, the total health expenditure on NCDs as a proportion of total health expenditure was 11% in the financial year 2017/18 [14].

### Study sites

This study was carried out in Busia and Trans Nzoia counties in Western Kenya, where Moi University and Moi Teaching and Referral Hospital through the AMPATH (Academic Model Providing Access to Healthcare) programme have partnered for several years with county governments to strengthen health systems across various care levels [35]. AMPATH provided the platform for implementing the Primary Care Project for Chronic Conditions (PIC4C) that was launched in 2018 to strengthen the primary care services for diabetes, hypertension, breast and cervical cancer in two counties in western Kenya [36]. The PIC4C model included (i) early case finding of people with hypertension, diabetes, cervical/ breast cancer at service level 1; (ii) structured referral to service providers at level 2 for confirmation of diagnosis and treatment initiation or referral to level 3 or 4 using structured protocols; (iii) initiation of treatment using structured treatment protocols and decision support tools at levels 2, 3 and 4; (iv) retention of patients in care supported by ongoing training of health workers at all care levels; (v) monitoring and evaluation supported by a health information system; and (vi) linking patients in care with the voluntary supa cover operated by the NHIF for sustainable health financing. This study was part of the PIC4C scale-up study—a larger study that aimed to evaluate how well PIC4C delivered on its intended objectives and to provide evidence to inform the scale-up of the PIC4C model for integrated NCD management in Kenya. Details of the PIC4C model and the larger study are published elsewhere [36]. Our study work package had qualitative and quantitative components with three objectives: (1) to measure the effectiveness of the NHIF national scheme benefit package in providing financial risk protection to individuals with hypertension/diabetes; (2) to examine the extent to which the NHIF national scheme benefit package is responsive to the needs of individuals with hypertension/diabetes; and (3) to examine how the provider incentives generated by provider payment arrangements of the NHIF national scheme benefit package influence equity, efficiency, and quality of care. This paper presents the findings of objective 1.

### Study design

The study employed a cohort study design to collect health expenditures, household expenditures, health-seeking behaviour and household sociodemographic data quarterly from 888 households. The cohort study recruited households to an exposure arm and a control arm and followed these households for a year (12 months). The **“exposure arm”** comprised of households that have at least one person with hypertension and/or diabetes and were enrolled in the NHIF national scheme while the “**control arm”** comprised of households that have at least one person with a chronic disease (hypertension and/or diabetes) and were not enrolled in any form of health insurance. The primary outcome of interest was the incidence of CHE.

### Study population

The study population were households in which at least one member had hypertension and/or diabetes in Busia and Trans Nzoia counties. Participants were recruited if they met the following criteria: (1) had a diagnosis of either hypertension or diabetes, (2) they were at least 18 years of age, (3) they resided within the study area and would be available for the next 12 months of follow-up, and (4) they were aware of their NHIF registration status.

### Sample size and sampling

We estimated that a minimum sample of 179 households per comparison group would have 80% power to detect a 15-percentage point difference in the proportion of OOP costs on healthcare as a share of total household expenditure, assuming a proportion of OOP costs of 40% in the control group, a design effect of 1.2, and a two-sided alpha level of 0.05. We estimated that up to 60% of the study participants could be potentially lost to follow-up due to refusals or the nature of the conditions they live with over one year and therefore, based on these assumptions, the sample size was adjusted upwards by 43% to a final sample size of 960. The sample size was distributed equally between the two groups with 480 in each.

Study participants were drawn from two sources. The first source was the AMPATH PIC4C chronic disease model screening database [36]. We first extracted a list of potential participants that met the study eligibility criteria, which were verified by telephone calls by research assistants. Due to insufficient numbers in the screening database, the second source was health facility registers where the PIC4C model was implemented. Contact details of these potential participants were obtained, and phone calls were made to establish eligibility. Participants enrolled from both screening database and health facility registered were beneficiaries of the PIC4C model. Stratified random sampling was used to select study participants. Eligible households were stratified by geography (two counties – Trans Nzoia and Busia) and type of NCD, and thereafter study participants were randomly selected from each of the strata. Table 1 outlines the distribution of the sample across geographical locations (counties), study arm, NCD and the households that were successfully recruited at baseline.

**Table 1:** Baseline household enrolment

	With NHIF			Without NHIF (Inactive & No NHIF)		
Condition	Target sample	Enrolled	% Attained	Target sample	Enrolled	% Attained
**Busia County**						
HTN	80	107	134%	80	102	128%
DM	80	43	54%	80	47	59%
Comorbid*	80	81	101%	80	83	104%
**Total**	**240**	**231**	**96%**	**240**	**232**	**97%**
**Trans-Nzoia County**						
HTN	80	80	100%	80	99	124%
DM	80	35	44%	80	49	61%
Comorbid*	80	78	98%	80	84	105%
**Total**	**240**	**193**	**80%**	**240**	**232**	**97%**

*Notes*: HTN – hypertension; DM – diabetes mellitus; *Both HTN and DM

### Data collection

Data were collected at participants’ homes by trained research assistants using electronic structured questionnaires based on the CommCare platform at enrolment and after every three months (follow-up) over one year. The baseline survey was conducted from March to May 2021 (n = 888 or 92% of the target sample size), and data for the second wave was collected from August to September 2021 (n = 769 or 87% of the baseline); data for the third wave was collected from November to December 2021 (n = 780 or 88% of the baseline) and data for the fourth wave was collected from March to April 2022 (n = 761 or 86% of the baseline). Research assistants were trained on data collection tools, interviewing techniques, and standard operating procedures for household interviews, including the informed consent process. Before the commencement of each subsequent wave, refresher training for the research assistants was provided. Quality control during data collection was assured by study coordinators who accompanied research assistants to households and verified the quality of data submitted at the end of each day. After the informed consent process and obtaining signed consent forms from eligible participants, the questionnaire was administered to the head of the household and/or the household member with a chronic disease. The structured questionnaire collected information on health-seeking events, general household expenditure, and healthcare expenditure, as well as other important household characteristic that were used to match households at analysis. These included county of residence, number of household members with chronic diseases, household size and household socioeconomic status.

### Cost measures

In each wave, self-reported direct medical costs (i.e., payments made to healthcare providers for services received) were collected for three types of care-seeking event. First, outpatient costs incurred in the last four weeks before the date of the interview were collected. Outpatient costs were made up of OOP costs for registration, consultation, diagnostic tests, and medicines. Second, routine care costs incurred by hypertension and/or diabetes patients when attending their scheduled clinic appointments were also collected. Routine care costs included medicine and health-related commodities expenses. Third, in-patient costs in the past 12 months were collected. In-patient costs included registration, medicines, consultation, surgical operation, daily bed rate, and diagnostic tests. Where there were challenges recalling disaggregated OOP cost items for outpatient and hospitalisation, participants were asked to report the total amount of money spent following a care-seeking event. OOP payments for each care-seeking episode were computed, less any health insurance reimbursements [37]. In addition, direct non-medical costs for transport (to and from healthcare providers) were collected for outpatient and inpatient care-seeking episodes. Cost estimates for each care-seeking episode were computed at the household level and annualised. Hospitalisation costs were however not annualised. NHIF premiums were not included in the cost computation because they are predictable [38].

To estimate household consumption expenditure, self-reported amounts spent on food weekly and non-food items monthly were collected and annualised. Data on self-reported expenditure on selected durable goods in the past year was also collected. To estimate average annual healthcare (outpatient, routine, and hospitalisation costs) and consumption expenditures across the four waves, the annual costs in each wave were summed up and divided by the number of times a household was successfully interviewed during the one-year follow-up. Majority of households (84%; n = 746) were interviewed four times, 11.6% (n = 103) were interviewed once, 3.7% (n = 33) were interviewed three times and 0.7% (n = 6) were interviewed two times.

### Data analysis

We carried out a descriptive analysis to summarize the data by computing means of total OOP costs incurred by sampled households to seek NCD care. The share of OOP expenditure in total household expenditure was computed as the proportion of total direct household healthcare expenditure in total household consumption expenditure. While there are various thresholds for measuring CHE, in this study households were considered to have incurred CHE if their annual OOP health expenditures exceeded 40% of their annual non-food expenditure (i.e. households’ capacity to pay) [39]. This threshold was chosen because it represents households’ true capacity to pay for healthcare after basic subsistence needs have been met [15]. In this paper, we analysed the incidence of CHE due to direct medical costs as well as direct non-medical costs (i.e., including transport costs). We also computed means for specific and overall costs that were reimbursed by NHIF. In addition, to assess hypertension and/or diabetes healthcare costs that are covered by the NHIF national scheme (i.e., “depth of cover”), we computed the mean proportion of overall hypertension and/or diabetes costs that were paid by NHIF among households that had an active NHIF enrolment. The Pearson’s chi-square, Kruskal Wallis and Mann-Whitney tests were used to test differences in OOP as a proportion of total annual household expenditure, and the incidence of incurring CHE, as appropriate.

Given that our exposure variable (household NHIF status) was likely to change over the 12 months study period, we classified households’ NHIF status across the four waves into *three* main groups. The first group was classified as households with *active NHIF*. These were households who remained enrolled in NHIF throughout the four waves and consistently paid their premium contributions, hence maintaining active membership and could therefore access healthcare services without OOP payment. The second group were classified as having *no NHIF.* These were households who were never enrolled in NHIF throughout the four waves or whose NHIF status was *inactive* (i.e., households that had NHIF cards but had not paid their monthly premiums and hence could not use the cards to access healthcare services) throughout the four waves. The third group were classified as *partial NHIF.* These were households who in one wave of the survey reported active NHIF enrolment and in another wave of the survey either reported inactive NHIF enrolment or no NHIF enrolment. However, to assess the effectiveness of NHIF in cushioning households against incurring CHE, we conducted both per-protocol and intention-to-treat analyses. That is, in the per-protocol analysis, households were categorised into *two groups*: ***active NHIF*** and ***no NHIF*** (the latter including no NHIF and inactive NHIF) based on whether they maintained NHIF enrolment status (active or inactive/no NHIF) across the four waves. By contrast, in the intention to treat analysis, we grouped households based on their NHIF status at baseline (i.e., active NHIF and no NHIF [no NHIF and inactive NHIF]) and analysed the likelihood of incurring CHE across the four waves. To estimate the NHIF attrition rate over 12 months, we computed the proportion of households that were active NHIF across the four waves compared to active NHIF households at baseline.

### Coarsened exact matching

To assess whether the level of financial protection differed between households enrolled in the NHIF national scheme and those not enrolled, we applied the coarsened exact matching (CEM) approach to match households using baseline characteristics [40]. We used CEM to account for the potential confounding influence of the following household pre-treatment (in our case by treatment we refer to NHIF status) variables: (1) household size, (2) the number of people with an NCD in a household, (3) county of residence, and (4) household socioeconomic status. The selection of these pre-treatment variables was informed by literature [15, 41–43]. The CEM approach has been described in detail in the literature [40, 44]. In brief, to control for potential confounding of “pre-treatment” covariates on the outcome of interest, CEM matches “treatment” and “non-treatment” households that are similar to them with regard to those covariates. The advantage of CEM over other approaches of matching observational data such as exact score matching (EM) and propensity-score matching (PSM) is that, unlike EM, it does not require exact similarity of matched observations by the selected covariates nor does it require matched observations to be balanced in terms of pre-treatment covariates as is the case for PSM [40, 44, 45]. Rather, in CEM, pre-treatment covariates are “coarsened” into categories depending on how they are distributed or their intuitive or natural divisions [40, 44, 45]. After matching, we fitted a conditional logistic regression model to assess the likelihood of experiencing CHE between NHIF-enrolled households and those not enrolled.

### Catastrophic costs inequality assessment

To explore inequality in CHE, we developed concentration curves and computed concentration indices of the level of OOP expenditure [46]. To facilitate this, we categorized the households in the sample into 5 socio-economic quintiles, (5 richest, 1 poorest) using the annual total household consumption expenditures as the measure of household wealth. The concentration curve plots the cumulative share of catastrophe (y-axis) against the cumulative share of households (x-axis), ranked from poorest to richest. The concentration curve lies above (below) the line of equality when catastrophic healthcare payments are concentrated among the poor (rich), with the gap between the concentration curve and the line of equality depicting the extent of inequality [46]. Defined as twice the area between the concentration curve and the line of equality, the concentration index (CI) lies between − 1 and 1, with a negative (positive) CI corresponding to a pro-poor (pro-rich) distribution of catastrophic healthcare payments [46, 47]. Additionally, the larger the absolute value of the CI, the greater the extent of inequality in catastrophic healthcare payments [46]. We applied the correction proposed by Erreygers in computing the CI given that our outcome was binary [48]. All statistical analyses were done using Stata version 15 (College Station, TX: Statacorp LLC) [49].

## Results

### Household NHIF enrolment and attrition rate

Table 2 presents households’ NHIF enrolment across the four waves. Almost half (46.9%) of households had active NHIF enrolment at baseline (wave 1) but this reduced to 10.9% after one year (wave 1–4). This means that the NHIF attrition rate was 76.7% over the follow-up period.

**Table 2:** Household NHIF enrolment over the study period

Study wave	Active NHIF n (%)	No NHIF n (%)	Inactive/Partial^†^ n (%)
Wave 1	416 (46.9)	280 (31.5)	192 (21.6)
Wave 2	355 (46.2)	263 (34.2)	151 (19.6)
Wave 3	368 (47.2)	272 (34.9)	140 (18.0)
Wave 4	375 (49.3)	238 (31.3)	148 (19.5)
Wave 1–4	97 (10.9)	113 (12.7)	678 (76.4)

^†^Partial applies to waves 1–4 only and includes households that changed from active to inactive/ no NHIF enrolment or vice versa over one year

### Healthcare seeking by facility type and NHIF status

**Fig. 1:**
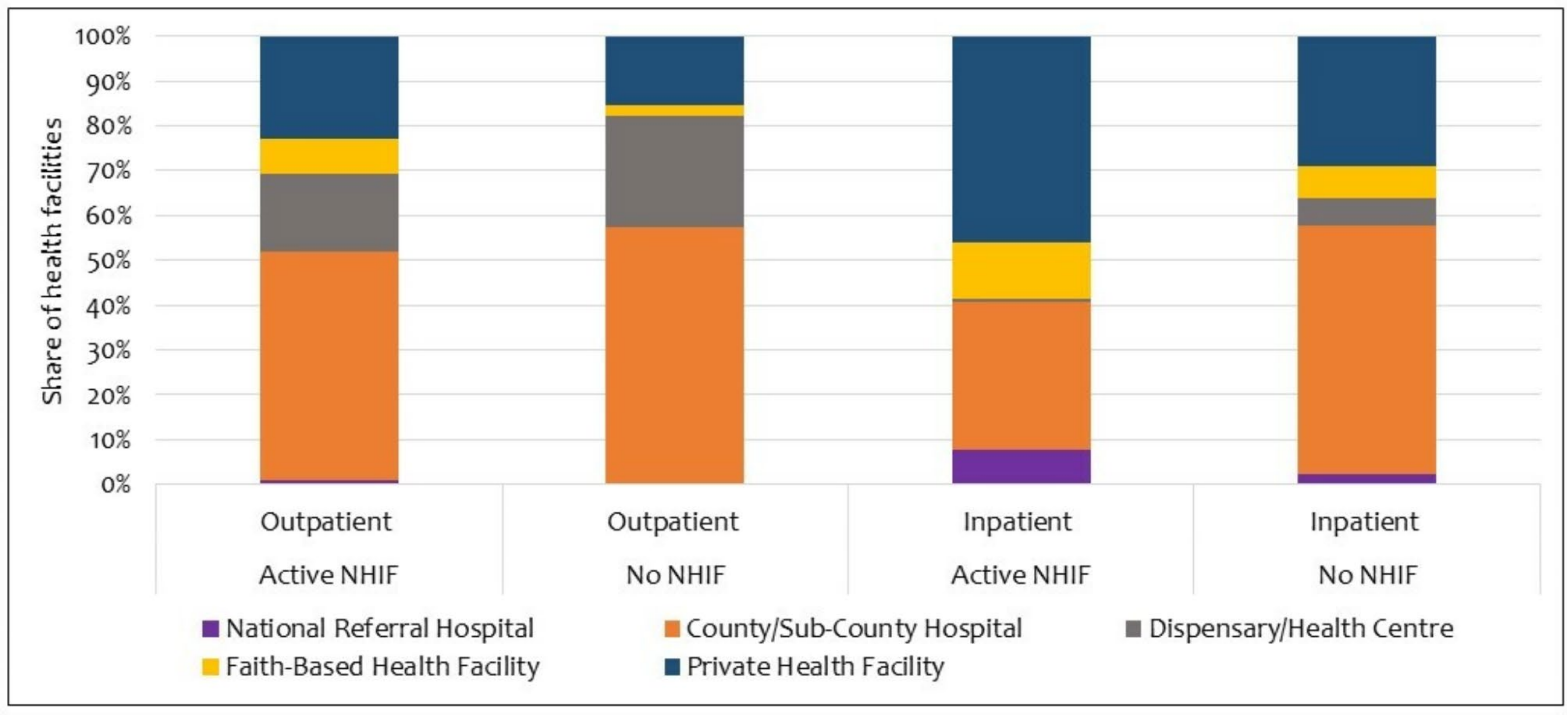
Healthcare facilities where care was sought by NHIF status

Figure 1 shows healthcare facilities where households sought care during the study period. Overall, across the four waves, households enrolled in the NHIF and those not enrolled mainly sought outpatient care from county/sub-county hospitals. However, a slightly higher share of households with no NHIF (57% [95% CI 41.9–66.4]) sought outpatient care from county/sub-county hospitals compared to households that had NHIF cover (51.2% [95% CI 44.0-58.5]). On the other hand, a higher share (46.1% [95% CI 36.5–55.6]) of households with NHIF cover sought care from a private facility for an inpatient admission compared to households with no NHIF (28.9% [95% CI 10.5–68.4]).

### OOP payment for direct healthcare and transportation costs

Table 3 outlines the estimates of average OOP expenditures split by wealth quintile, NHIF status and county. The mean annual total OOP payment for outpatient services was KES 39,524 (USD 315), KES 17,626 (USD 141) for routine care and KES 26,210 (USD 209) for inpatient admissions. Additionally, households spent on average KES 9,922 (USD 79) on transport to and from health facilities for outpatient and inpatient care. Richer households incurred higher OOP (healthcare, transport and overall) costs to access healthcare services compared to poorer households. Further, the mean annual total healthcare costs were higher for households that were not enrolled in the NHIF throughout the study period compared to households that were enrolled. Other than transport costs, healthcare costs for outpatient services, inpatient admission, routine care, and overall costs, were generally higher in Trans Nzoia County compared to Busia County.

**Table 3:** Mean annual OOP payments (KES) by income quintiles, NHIF enrolment, NCD status and county*

	Outpatient (SE)	Routine care (SE)	Inpatient (SE)	Total Healthcare costs (SE)	Transport (SE)	Overall (Healthcare + Transport) (SE)
Wealth quintile						
Quintile 1 (poorest)	29,757 (4,166)	18,442 (3,467)	13,294 (3,435)	38,962 (4,495)	9,583 (1,733)	46,439 (5,060)
Quintile 2	33,397 (3,603)	11,978 (1,776)	10,453 (2,875)	38,441 (3,624)	9,293 (885)	46,143 (3,996)
Quintile 3	33,291 (4,511)	13,842 (2,679)	24,197 (10,782)	45,330 (6,162)	9,184 (1,267)	53,418 (6,790)
Quintile 4	40,597 (7,817)	13,811 (2,436)	39,537 (13,122)	57,689 (10,757)	10,687 (1,459)	66,574 (10,687)
Quintile 5 (richest)	60,999 (15,328)	28,976 (10,044)	43,690 (19,064)	80,656 (16,683)	10,894 (1,321)	86,806 (16,076)
**NHIF enrolment (Wave 1 to 4)**						
Active NHIF	33,803 (7,660)	12,587 (4,236)	42,769 (22,847)	42,503 (8,752)	11,886 (2,234)	49,077 (8,323)
No NHIF (Inactive + No NHIF)	44,865 (11,221)	8,706 (1.712)	9,754 (2,256)	48,796 (10,846)	12,999 (3,096)	58,345 (11,341)
Partial NHIF^†^	39,434 (4,266)	18,883 (2,749)	27,096 (5,816)	53,576 (5,052)	9,210 (552)	61,368 (5,096)
**NCD status**						
Comorbid	45,421 (7,351)	15,135 (2,050)	30,560 (8,511)	58,263 (7,865)	9,688 (812)	66,095 (7,920)
Diabetes	30,413 (3,212)	24,478 (8,247)	14,183 (6,452)	41,823 (6,070)	9,564 (1,197)	49,235 (6,375)
Hypertension	36,501 (4,592)	17,690 (4,048)	26,171 (7,920)	49,075 (6,002)	10,316 (1,108)	57,030 (5,975)
**County**						
Busia	36,596 (3,856)	14,878 (1,867)	19,516 (4,849)	46,538 (4,374)	10,874 (1,071)	55,070 (4,593)
Trans Nzoia	42,677 (6,444)	20,563 (4,504)	34,075 (9,305)	57,915 (7,521)	8,880 (482)	64,920 (7,430)
**Total**	39,524 (3,690)	17,626 (2,383)	26,210 (5,028)	52,013 (4,274)	9,922 (606)	59,766 (4,281)

*Only households reporting OOP payments for care-seeking events

† Includes households that changed from active to inactive/no NHIF enrolment or vice versa over one year

### OOP payment by facility type and ownership

**Fig. 2:**
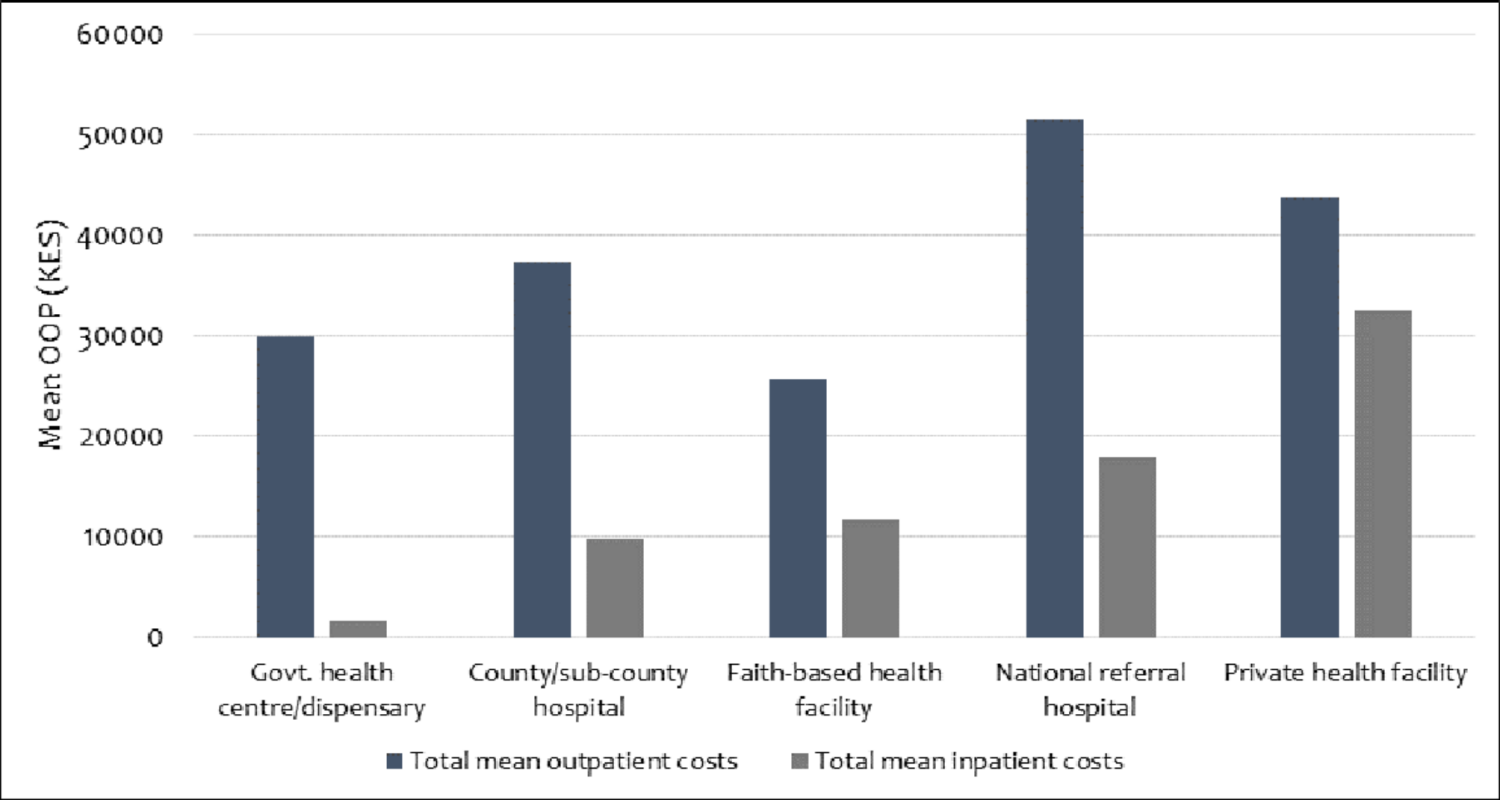
Mean annual out-of-pocket costs by facility type and ownership

Outpatient costs were highest for care provided in national referral hospitals and private health facilities (Fig. 2). Of note, annual outpatient costs were higher than inpatient costs across all types of health facility. In addition, in public sector facilities managed by county governments, care-seeking costs were highest in public hospitals compared to lower-level health facilities (Fig. 2).

### Level of household OOP healthcare expenditure

Overall, for total annual costs during the study period, we found strong evidence that NHIF-enrolled households spent a lower proportion (12.4%) of their total annual household expenditure on healthcare than households not enrolled in the NHIF (23.2%) (*p*-value = 0.004) (Table 4). Also, we found strong evidence that the poorest households spent a higher share (26.5%) of their total annual household expenditure on healthcare costs compared to the richest households (13.1%) for all care-seeking episodes (*p* < 0.001). In addition, we did not find strong evidence that there was a difference in the share of household expenditure spent on health between Busia and Trans Nzoia counties and across household NCD status (*p* > 0.05).

**Table 4:** Annual out-of-pocket expenditures as a proportion of total annual household expenditures

	Annual outpatient costs as a % Total household expenditure (95% CI)	Annual routine care cost as a % of Total household expenditure (95% CI)	Annual inpatient cost as a % of Total household expenditure (95% CI)	Annual total healthcare cost as a % of Total household expenditure (95% CI)	Annual transport cost as a % of Total household expenditure (95% CI)	Annual total costs (healthcare + transport) as a % of Total household expenditure (95% CI)	*p-*value
Socioeconomic status							
Quintile 1 (Poorest)	22.2 (11.7–32.8)	9.1 (5.9–12.3)	7.0 (3.5–10.6)	26.5 (16.3–36.8)	6.2 (3.9–8.6)	31.4 (20.3–42.4)	**< 0.001** ^†^
Quintile 2	12.3 (9.7–14.9)	4.4 (3.1–5.6)	3.7 (1.8–5.7)	14.1 (11.5–16.7)	3.4 (2.8–4.1)	17.0 (14.1–19.8)	
Quintile 3	9.9 (7.2–12.5)	4.1 (2.5–5.8)	7.3 (0.7–13.9)	13.5 (9.9–17.1)	2.7 (2.0-3.5)	15.9 (11.9–19.9)	
Quintile 4	9.4 (6.0-12.8)	3.2 (2.1–4.3)	9.2 (3.1–15.3)	13.4 (8.7–18.1)	2.6 (1.9–3.2)	15.5 (10.8–20.3)	
Quintile 5 (Richest)	10.2 (4.8–15.7)	4.0 (1.6–6.4)	6.7 (1.2–12.2)	13.1 (7.3–18.8)	1.8 (1.3–2.2)	14.1 (8.5–19.6)	
**NHIF enrolment (Wave 1 to 4)**							
Active NHIF	10.9 (4.9–16.9)	2.4 (1.0-3.8)	8.5 (-1.0-18.1)	12.4 (6.4–18.4)	4.3 (2.5–6.2)	15.1 (8.9–21.4)	**0.004** ^‡^
No NHIF (Inactive + No NHIF)	22.2 (5.5–38.9)	2.6 (1.4–3.8)	3.2 (1.0-5.3)	23.2 (7.0-39.3)	5.8 (2.1–9.6)	27.4 (10.4–44.4)	
**NHIF enrolment (Wave 1 to 4)**							
Active NHIF	10.9 (4.9–16.8)	2.4 (1.1–3.7)	8.5 (-0.7-17.8)	12.4 (6.4–18.3)	4.3 (2.5–6.2)	15.1 (8.9–21.4)	**0.002** ^†^
No NHIF (Inactive + No NHIF)	22.2 (5.6–38.8)	2.6 (1.5–3.8)	3.2 (1.1–5.2)	23.2 (7.1–39.2)	5.8 (2.1–9.6)	27.4 (10.5–44.3)	
Partial NHIF*	11.6 (9.6–13.7)	5.3 (4.2–6.4)	7.4 (4.610.2)	15.5 (13.1–17.9)	2.8 (2.5–3.2)	18.0 (15.5–20.5)	
**Wave 1 NHIF status**							
Active NHIF	12.0 (9.3–14.7)	5.0 (3.6–6.3)	7.8 (3.6–12.1)	15.4 (12.1–18.6)	3.6 (2.9–4.2)	18.0 (14.7–21.4)	0.289^‡^
No NHIF (Inactive + No NHIF)	13.4 (9.3–17.4)	4.9 (3.5–6.2)	6.3 (3.6-9.0)	16.7 (12.6–20.8)	3.2 (2.3-4.0)	19.4 (15.0-23.8)	
**NCD status**							
Comorbid	14.8 (9.6–20.1)	4.8 (3.2–6.4)	7.9 (4.0-11.9)	18.4 (13.0-23.7)	3.3 (2.6-4.0)	21.1 (15.4–26.8)	0.924^†^
Diabetes	10.3 (6.9–13.6)	6.7 (2.9–10.6)	3.8 (0.7-7.0)	13.3 (9.4–17.2)	3.0 (2.2–3.8)	15.6 (11.3–19.9)	
Hypertension	11.4 (8.9–13.9)	4.5 (3.4–5.6)	7.2 (3.4–11.1)	14.6 (11.6–17.6)	3.5 (2.5–4.6)	17.4 (14.2–20.7)	
**County**							
Busia	11.0 (8.9–13.2)	4.6 (3.4–5.8)	4.6 (3.4–5.8)	14.1 (11.6–16.6)	3.6 (2.7–4.5)	17.0 (14.2–19.8)	0.643^‡^
Trans Nzoia	14.5 (9.8–19.2)	5.2 (3.7–6.8)	5.2 (3.7–6.8)	18.2 (13.3–23.1)	3.1 (2.5–3.5)	20.7 (15.6–25.7)	

* Includes households that changed from active to inactive/no NHIF enrolment or vice versa over one year, † Kruskal Wallis test, ‡ Mann-Whitney test

Notes: significant *p*-values at 5% level of significance in bold

### Incidence of catastrophic healthcare expenditure

The proportion of households incurring CHE was 17.9% for the entire sample (i.e., regardless of study arm). The inclusion of transport costs increased the proportion of households incurring CHE to 23% (Table 5). We did not find strong evidence (*p-*value = 0.365) that the incidence of CHE was lower in households with active NHIF (14.3%) compared with households with no NHIF (19.6%). However, we observed strong evidence for difference across the wealth quintiles, with 35.8% of the poorest households incurring CHE compared to the richest households (10%) (*p-*value < 0.001). There was no strong evidence that the incidence of CHE was different by county or NCD condition (Table 5). There was strong evidence that the incidence of CHE was higher for outpatient services 13% (95% CI: 10.8–15.5) compared to inpatient admission 7.6% (95% CI: 4.5–11.9) (*p*-value = 0.001).

**Table 5:** Incidence of catastrophic healthcare costs (CHE) in Busia and Trans Nzoia Counties

Variables	Healthcare costs		Healthcare + transport costs		
	n	% (95% CI)	n	% (95% CI)	p-value*
Socioeconomic status					
Quintile 1 (poorest)	162	35.8 (28.4–43.7)	171	43.3 (35.7–51.1)	**0.000**
Quintile 2	168	20.2 (14.4–27.1)	175	25.7 (19.4–32.9)	
Quintile 3	170	11.8 (7.3–17.6)	174	15.5 (10.5–21.8)	
Quintile 4	167	12.0 (7.4–17.9)	172	18.0 (12.6–24.6)	
Quintile 5 (richest)	160	10.0 (5.8–15.7)	170	12.4 (7.8–18.3)	
**NHIF enrolment (Wave 1 to 4)**					
Active NHIF	77	14.3 (7.4–24.1)	88	18.2 (10.8–27.8)	0.365
No NHIF (Inactive + No NHIF)	92	19.6 (12.0-29.1)	99	28.3 (19.7–38.2)	
**NHIF enrolment (Wave 1 to 4)**					
Active NHIF	77	14.3 (7.4–24.1)	88	18.2 (10.8–27.8)	0.283
No NHIF (Inactive + No NHIF)	92	19.6 (12.0-29.1)	99	28.3 (19.7–38.2)	
Partial NHIF^†^	658	18.1 (15.2–21.2)	675	22.8 (19.7–26.2)	
**Care seeking**					
Outpatient	806	13.0 (10.8–15.5)	854	16.5 (14.1–19.2)	**0.001**
Routine	300	5.3 (4.5–11.9)	—	—	
Inpatient	224	7.6 (4.5–11.9)	291	6.2 (3.7–9.6)	
**NCD status**					
Comorbid	350	18.3 (14.4–22.7)	361	23.0 (18.7–27.7)	0.954
Diabetes	129	17.8 (11.7–25.5)	136	22.1 (15.4–30.0)	
Hypertension	345	17.4 (13.5–21.8)	362	23.2 (19.0-27.9)	
**County**					
Busia	429	16.8 (13.4–20.7)	451	23.1 (19.2–27.2)	0.386
Trans Nzoia	398	19.1 (15.4–23.3)	411	22.9 (18.9–27.2)	
**Overall**	**827**	**17.9 (15.4–20.7)**	**862**	**23.0 (20.3–25.9)**	

† Includes households that changed from active to inactive NHIF enrolment or vice versa over one year

Notes: significant p-values at 5% level of significance in bold, *Pearson’s Chi-squared test

### Socioeconomic inequality in catastrophic healthcare expenditure

**Fig. 3:**
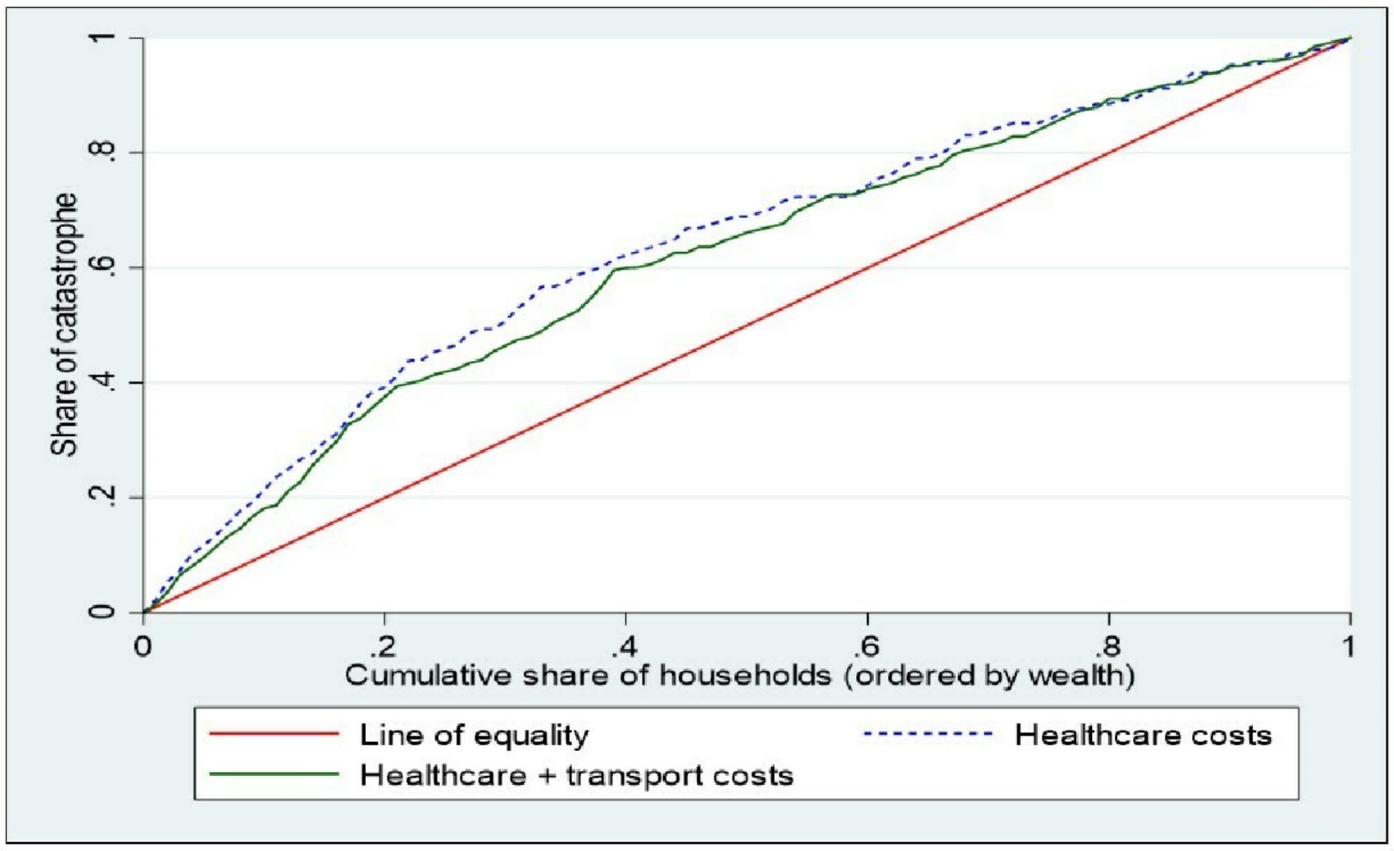
Concentration curves of Catastrophic healthcare costs due to healthcare and transport costs

The concentration curves in Fig. 3 suggest that the incidence of catastrophic healthcare costs (due to healthcare costs, and healthcare costs combined with transport costs) are concentrated among the poor. These findings are confirmed by the negative values of the concentration indices for CHE (Table 6). There was strong evidence that CHE due to healthcare costs was concentrated among the poor (CI: -0.190, *p* < 0.001).

**Table 6:** Concentration indices for incurring catastrophic healthcare costs

Cost category	n	Concentration index	Std. Error	*p-*value
Healthcare cost	827	-0.190	0.029	0.000
Healthcare + transport cost	862	-0.222	0.032	0.000
Transport cost	857	-0.448	0.012	0.000

### Depth of NHIF cover

**Fig. 4:**
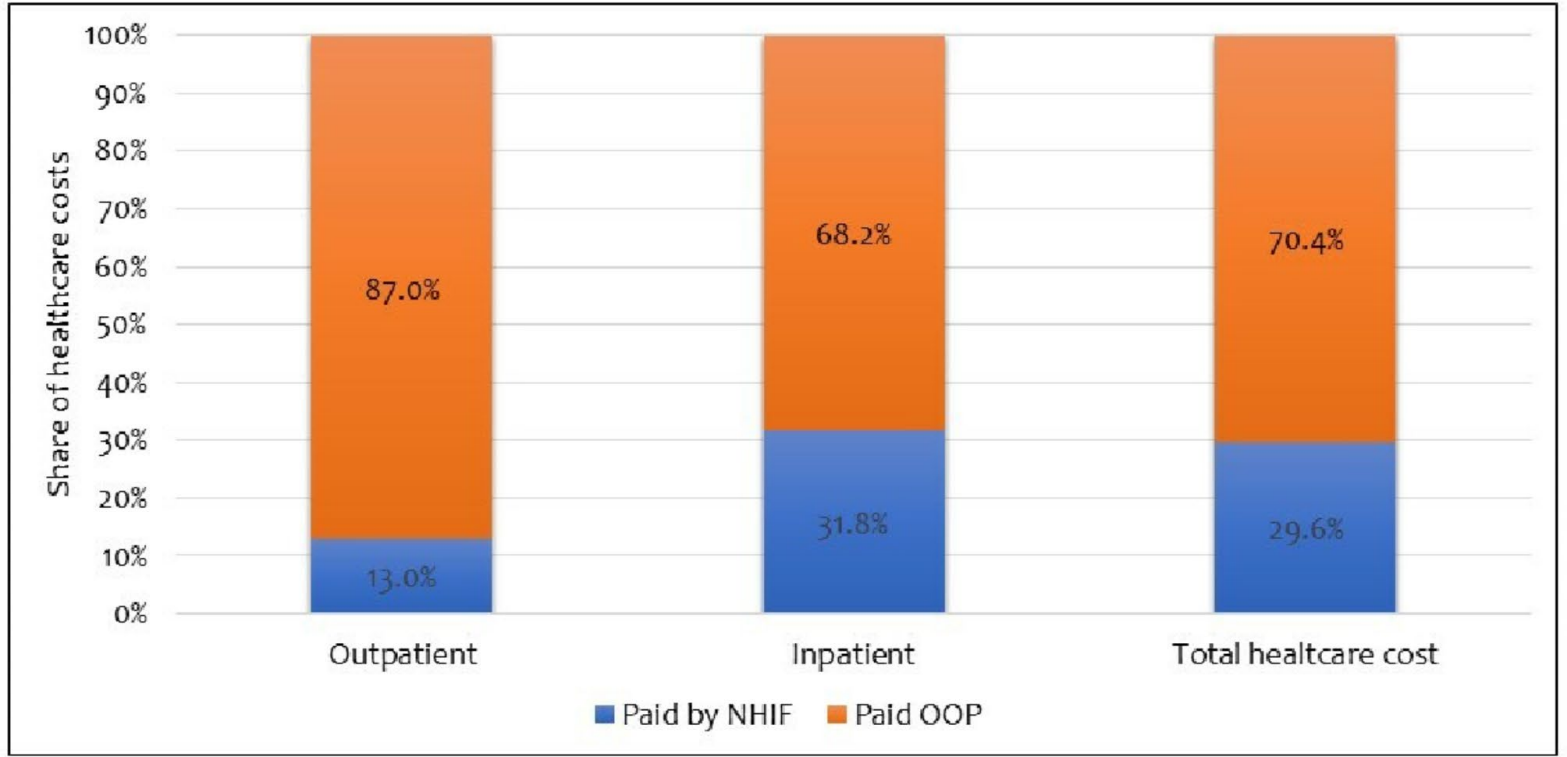
Proportion of healthcare costs covered by NHIF among NHIF active households

Among households with active NHIF, the depth of NHIF cover (i.e., the proportion of healthcare costs paid by NHIF) was more than double (31.8%) for hospitalisation costs compared to outpatient costs (13%) (Fig. 4). Moreover, NHIF covered only 29.6% of total healthcare costs among households with active NHIF (Fig. 4).

### NHIF effectiveness in cushioning households against CHE

After matching, we did not find strong evidence from the conditional logistic regression that the odds of incurring catastrophic health expenditure between NHIF households and non-NHIF households is different (Table 7). This was the case for both the per-protocol (OR 0.67 [95% CI 0.22–2.02]) and intention-to-treat analysis (OR 0.99 [0.67–1.46]), and whether or not transport costs were considered.

**Table 7:** Conditional logistic regression model for likelihood of incurring CHE [incurred CHE = 1; otherwise = 0] over one year period

NHIF status	CHE due to OOP healthcare costs as the dependent variable			CHE due to OOP healthcare and transport costs as the dependent variable		
	Odds Ratio (95% CI)	Std. Error	*p-*value	Odds Ratio (95% CI)	Std. Error	*p-*value
No NHIF (Inactive + No NHIF) (wave 1–4) (Ref)						
†Active NHIF (wave 1–4)	0.67 (0.22–2.02)	0.38	0.47	0.69 (0.29–1.66)	0.31	0.41
No NHIF (Inactive + No NHIF) (wave 1–4) (Ref)						
‡Active NHIF (wave 1–4)	0.99 (0.67–1.46)	0.20	0.96	0.88 (0.62–1.24)	0.16	0.47

^†^Per protocol analysis; ^‡^Intention to treat analysis

## Discussion

This study presents an analysis of the effectiveness of NHIF in offering financial protection to households with hypertension and/or diabetes patients using a one-year cohort study design. First, we found strong evidence that households with no NHIF spent a higher share of their total annual household expenditure on healthcare compared to households with NHIF. However, we did not find strong evidence that having an active NHIF cover, that entitles one to the national scheme benefit package, protects households that have an individual living with hypertension and/or diabetes from incurring catastrophic health expenditures. Similar findings have been reported elsewhere in China [42, 43], Korea [50] and Vietnam [41]. For example, to reduce the financial burden associated with seeking hypertension and diabetes care in rural China, the benefits package and population coverage of the medical scheme were expanded. Nevertheless, a study by Liu et al. [42] that assessed the effect of these policies found a significant increase in CHE due to medical expenditures for outpatient services.

The inadequacy of the NHIF national scheme in providing financial risk protection to households with individuals living with an NCD is perhaps explained by the second finding from this study, that the national scheme provided a low depth of cover (29.6%), implying that household still had to pay for over 70% of their healthcare costs out of pocket. A recent study in Kenya has revealed that NHIF offers inadequate financial protection to households due to poor benefits package design [51]. These findings are corroborated by those of a qualitative assessment that found that important services sought by individuals living with hypertension and/or diabetes, such as medicines and laboratory monitoring tests, were not paid for by the NHIF [52]. This could also be because of the NHIF’s provider payment mechanisms that do not specifically pay for key services utilized by people living with hypertension and diabetes. Specifically, the NHIF pays for outpatient services using a capitation mechanism that bundles all outpatient services without risk adjustment. For instance, it is expected that routine monitoring and medicine prescription costs are covered by the capitation payment that is standardized across all facilities of the same level of care and ownership without adjustment for the case mix of the catchment population. Given that the capitation rate is not risk-adjusted to account for the higher costs for chronic diseases, the current capitation mechanism does not adequately compensate health facilities for providing care to patients with NCDs potentially incentivising health providers to either balance bill or deny patients certain NCD services (e.g., prescription medicines and monitoring services). A previous study reported that health providers in Kenya preferred a capitation mechanism that included a limited number of health services under the capitation payment rate [53]. There is also some evidence that individuals with an active NHIF cover were more likely to use private health facilities compared to those without active NHIF. It is likely that this increases their exposure to OOP due to the higher cost of care in the private health sector, attenuating the effect of the NHIF in reducing OOP. This preference for private healthcare facilities could be because of perceptions of differences in quality of care which is reported elsewhere [52].

Third, the incidence of CHE reported in the study due to direct medical costs was relatively high (17.9%) and increased to 23% when transport costs were included. Previous studies have shown that non-medical costs like transport adds to the cost burden experienced by patients in seeking care [15–17, 54]. In Kenya for instance, it has been shown that transport costs represented 38% and 23% of direct costs to access hypertension and diabetes care, respectively [16, 17]. Therefore, our study is aligned with the evidence base that transport costs constitute a significant financial barrier to accessing healthcare services for NCDs in Kenya [15].

Fourth, the NHIF attrition rate over one year observed in this study was high (76.3%). This compromises the effectiveness of the NHIF, a contributory scheme, as a financial risk protection mechanism. This is explained by the fact that over 80% of the Kenyan population is in the informal labour market, expected to make (*de facto*) voluntary premium contributions to the NHIF [55]. There is overwhelming global evidence that voluntary health insurance contributions are associated with high adverse selection and attrition of members [56]. We found further insights about this situation in a parallel qualitative study with hypertension and diabetes patients [52]. Respondents highlighted barriers to enrolment and retention in the NHIF that include high levels of poverty, penalties associated with defaulting payment, and the current monthly NHIF premium rates (USD 4) that were perceived as unaffordable, especially to rural dwellers [52].

Fifth, we found CHE to be disproportionately concentrated among the poorest households compared to the wealthiest households. Other studies have found that the poorest NCD households that cannot cope with CHE may simply opt out of care leading to a higher risk of worsening health conditions and potentially increased financial burden [57]. As a result, it is likely that the true impact of OOP on low-income households may be underestimated.

Sixth, this study also assessed where households sought healthcare services the most throughout the study period. We found that county and sub-county hospitals were the most common source for outpatient services for households enrolled in NHIF and those that are not and that costs were higher at this level compared to primary healthcare (PHC) facilities. There is evidence that households tend to forego care from nearly accessible PHC facilities in preference for higher-level facilities due to poor quality care such as an erratic supply of medicines and other essential commodities [15, 51]. Bypassing more accessible PHC facilities is likely to increase transportation costs as was observed in this study. Moreover, whereas the PIC4C model aimed to ensure initiation of diabetes and hypertension treatment, at levels 2, 3 and 4, it is interesting to note that despite this, care was mostly sought from higher level facilities.

We draw several recommendations from this study’s findings. First, for the NHIF to enhance its effectiveness in providing financial risk protection to households with individuals living with hypertension and/diabetes, its national scheme benefit package should be reviewed to include services that are commonly consumed by persons living with hypertension and/or diabetes. Second, the NHIF provider payment mechanisms should be reformed in one of two ways. The capitation rates could be risk-adjusted to better represent the costs to providers for caring for these patients and mitigate their tendency to underprovide for persons living with hypertension and/diabetes. Another option would be to unbundle specific essential hypertension and/diabetes services from the existing capitation payment and pay for them separately. This includes costs for routine monitoring and prescription medicines. Second, county governments should invest in enhancing the capacity of PHC facilities to provide healthcare services to persons living with hypertension and/or diabetes. This will increase the geographical accessibility of services but also reduce costs incurred to access care. Third, to address the affordability of NHIF premiums and attrition, the government of Kenya should include individuals in the informal labour market in its health insurance subsidy scheme and expand the population coverage of this scheme to cover more poor and informal sector poor households. Also, given the potential role that quality-of-care plays in the observed preference for private healthcare facilities among individuals with an active NHIF cover, efforts to scale up the PIC4C model in Kenya should consider including a component for strengthening the structural quality of care, e.g., availability of medicines and essential diagnostic services of public healthcare facilities.

Whereas previous studies have mainly relied on cross-sectional designs, to our knowledge, our study is the first to use a longitudinal study design to assess the effectiveness of NHIF in offering financial protection to households with individuals living with hypertension and/or diabetes. The study is further strengthened by the collection of repeated measures (in four rounds of data collection). Thus, we were able to adequately capture the dynamics in health expenditures and insurance coverage over a long period. However, this study also had limitations. First, the arguably low number of diabetic patients recruited at baseline (due to low prevalence) and the high NHIF attrition rate over the one-year follow-up possibly underpowered our study especially in the per protocol analysis. Second, whilst quality healthcare is a critical UHC goal, we did not assess the level of quality for diabetes and hypertension healthcare services in this study. Although we did not find strong evidence of reduction of CHE among NHIF-enrolled households, it might be the case that the quality of care improved for this group due to improved access given the attenuated financial risk protection effect of the NHIF. This should be explored in future research. Third, we intended to include cervical and breast cancer patients in this study. However, due to difficulty in getting a complete and linked data from AMPATH’s surveillance system, cancer patients were excluded. Therefore, future research should consider assessing the effectiveness of health insurance schemes in ensuring financial protection for cancer patients. In addition, given that household health expenditures were self-reported, there is a risk of recall bias. Nevertheless, our study was conducted quarterly thus reducing the period for recall. Lastly, our study was only limited to two counties and may not be generalizable to the entire country. Specifically, we note that participants who benefitted from the PIC4C model are different from other diabetes/hypertension patients in the country due to exposure to early screening and linkage to care through structured referrals as well as the voluntary supa cover operated by NHIF.

## Conclusion

Non-communicable diseases have emerged as a key health burden for the Kenyan population. NCDs are also associated with a higher risk of catastrophic health expenditures. Kenya has positioned the NHIF as a key mechanism for purchasing health services and providing financial risk protection to the population. This study did not find strong evidence that the NHIF national scheme membership protects households with individuals living with hypertension and/or diabetes from incurring catastrophic health expenditures. As part of country reforms to tackle NCDs, and to accelerate progress towards UHC, reforms should review the NHIF benefit package and the capacity of the primary health care system to deliver care to enhance the responsiveness of the health system to meet the healthcare needs of people living with hypertension and/or diabetes.

## Data Availability

The data used in the analysis is available upon reasonable request from the corresponding author (RO) and subject to institutional data governance committee policies.

## Abbreviations

AMPATH: Academic Model Providing Access to Healthcare
CEM: Coarsened Exact Matching
CHE: Catastrophic Health Expenditure
CI: Concentration Index
DM: Diabetes Mellitus
EM: Exact Matching
GDP: Gross Domestic Product
HTN: Hypertension
KES: Kenya Shillings
LMICs: Low-and-Middle-Income Countries
NCDs: Non-Communicable Diseases
NHIF: National Health Insurance Fund
OOP: Out-of-pocket
PIC4C: Primary Care Project for Chronic Conditions
OR: Odds Ratio
PHC: Primary Healthcare
PSM: Propensity Score Matching
SE: Standard Error
UHC: Universal Health Coverage
USD: United States Dollar
